# Gene expression network analysis provides potential targets against SARS-CoV-2

**DOI:** 10.1038/s41598-020-78818-w

**Published:** 2020-12-14

**Authors:** Ana I. Hernández Cordero, Xuan Li, Chen Xi Yang, Stephen Milne, Yohan Bossé, Philippe Joubert, Wim Timens, Maarten van den Berge, David Nickle, Ke Hao, Don D. Sin

**Affiliations:** 1 Centre for Heart Lung Innovation, University of British Columbia, Vancouver, BC Canada; 2 Division of Respiratory Medicine, Faculty of Medicine, University of British Columbia, Vancouver, BC Canada; 3 Faculty of Medicine and Health, University of Sydney, Sydney, NSW Australia; 4 Institut Universitaire de Cardiologie et de Pneumologie de Québec, Université Laval, Québec City, QC Canada; 5 Department of Pathology and Medical Biology, University of Groningen, University Medical Center Groningen, Groningen, The Netherlands; 6 Department of Pulmonary Diseases, University of Groningen, University Medical Center Groningen, Groningen, The Netherlands; 7 Merck Research Laboratories, Genetics and Pharmacogenomics, Boston, MA USA; 8 Department of Genetics and Genomic Sciences and Icahn Institute for Data Science and Genomic Technology, Icahn School of Medicine At Mount Sinai, New York, NY USA

**Keywords:** Genetics, Pathogenesis, Risk factors

## Abstract

Cell entry of SARS-CoV-2, the novel coronavirus causing COVID-19, is facilitated by host cell angiotensin-converting enzyme 2 (ACE2) and transmembrane serine protease 2 (TMPRSS2). We aimed to identify and characterize genes that are co-expressed with *ACE2* and *TMPRSS2*, and to further explore their biological functions and potential as druggable targets. Using the gene expression profiles of 1,038 lung tissue samples, we performed a weighted gene correlation network analysis (WGCNA) to identify modules of co-expressed genes. We explored the biology of co-expressed genes using bioinformatics databases, and identified known drug-gene interactions. *ACE2* was in a module of 681 co-expressed genes; 10 genes with moderate-high correlation with *ACE2* (r > 0.3, FDR < 0.05) had known interactions with existing drug compounds. *TMPRSS2* was in a module of 1,086 co-expressed genes; 31 of these genes were enriched in the gene ontology biologic process ‘receptor-mediated endocytosis’, and 52 *TMPRSS2-*correlated genes had known interactions with drug compounds. Dozens of genes are co-expressed with *ACE2* and *TMPRSS2*, many of which have plausible links to COVID-19 pathophysiology. Many of the co-expressed genes are potentially targetable with existing drugs, which may accelerate the development of COVID-19 therapeutics.

## Introduction

The novel coronavirus SARS-CoV-2 is responsible for the current COVID-19 pandemic, which to date has infected over 44 million people and led to 1.1 million deaths worldwide^1^. Despite global efforts to study existing and novel therapeutics against the disease, there are currently no genuinely effective treatments. A better understanding of the biology of the virus and the host response to infection is essential if we are to improve clinical outcomes in COVID-19.

A number of genes expressed in lung tissue that are relevant to COVID-19 have been identified. These include genes that encode viral receptors (including ACE2 and BSG)^2–4^, host cell proteases (TMPRSS2, ADAM17 and FURIN)^2,3,5^, and factors related to the host response to infection (particularly the type I interferon response)^6,7^. However, identifying such genes experimentally is problematic since obtaining lung tissue samples from large numbers of COVID-19 patients is labour intensive, expensive, and may not be rapid enough to meet the urgency of the current pandemic. Moreover, the influence of severe lung inflammation may distort the expression of genes that are relevant to SARS-CoV-2 susceptibility or the earliest stages of COVID-19. However, existing large data sets of lung gene expression – derived from lung tissue samples that have been carefully collected, processed and analysed –provide an opportunity to explore genes relevant to the disease without the burden of collecting new samples under pandemic conditions.

Gene coexpression network analysis identifies genes whose expression is highly coordinated across the transcriptome^8^, suggesting that they are active simultaneously and thus active in the same biological process. Network analysis can be used for a number of purposes, including functional exploration and prioritisation of candidate genes. It is therefore a useful discovery tool in situations where little is known about the disease of interest, and thus may be of use in the fight against COVID-19.

We have previously reported on a large gene expression data set known as the Lung eQTL Study^9^. This study collected non-cancerous lung tissue samples from over 1,000 participants across three centres, applied coordinated processing and quality control standards, and collected detailed clinical information to accompany each specimen. This data set is therefore ideal for identifying genes that may be relevant to COVID-19, and examining the effects of various clinical risk factors on the expression of these genes.

Here we used the weighted gene coexpression network analysis (WGCNA)^8^ platform to identify genes connected to two important components of COVID-19 biology: *ACE2*, which encodes the putative SARS-CoV-2 receptor^3,4^; and *TMPRSS2*, which encodes a protease that primes the SARS-CoV-2 spike protein and facilitates entry of the virus into cells^3,10^. We used bioinformatics databases to explore the functional roles of coexpressed genes, and identified genes within these networks with known gene-drug interactions. Finally, we performed differential gene expression analysis on the most highly-correlated genes according to known clinical risk factors for severe COVID-19. These results may help direct future research on COVID-19 pathophysiology, drug repurposing, or the development of novel therapeutics.

## Results

**Figure 1:**
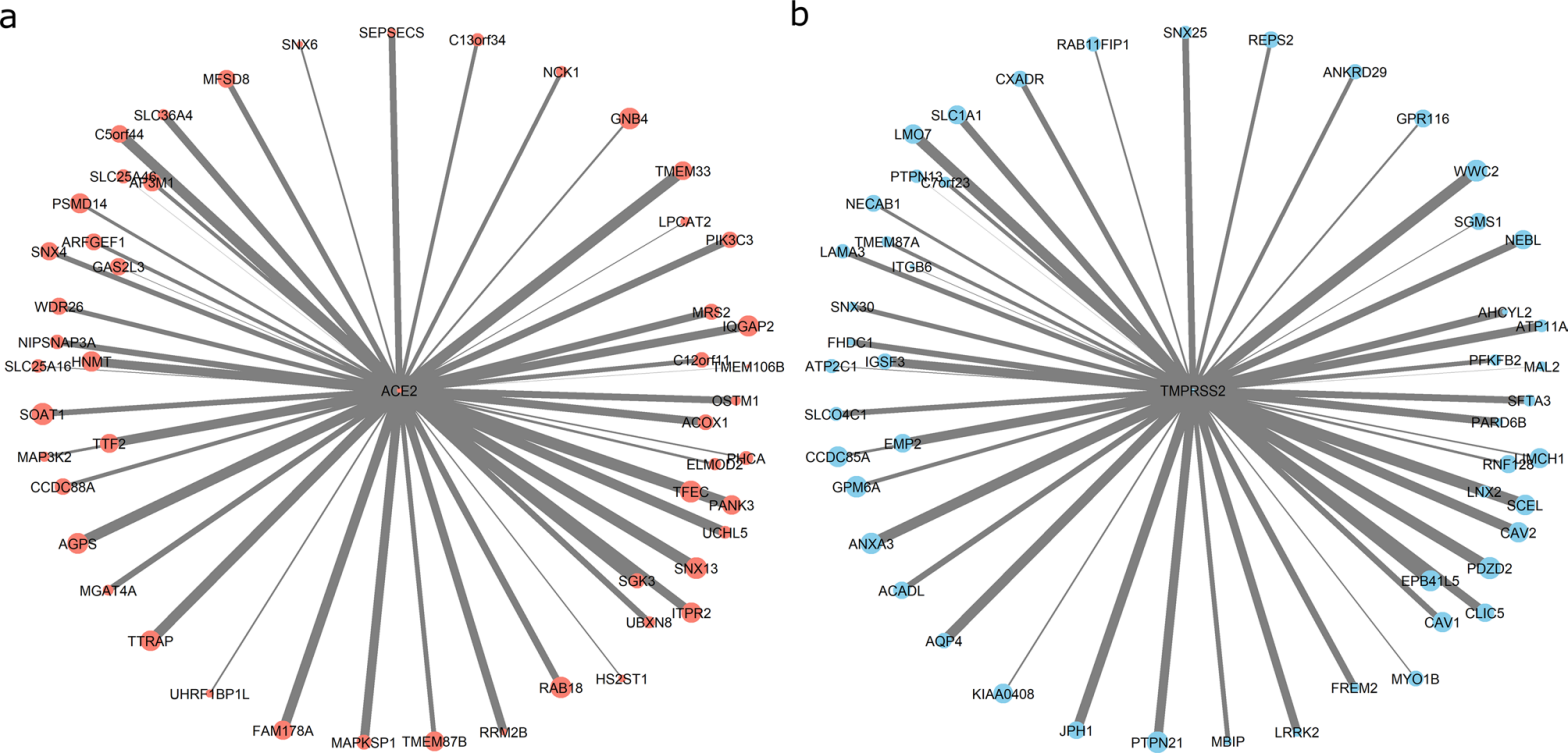
*ACE2* and *TMPRSS2* coexpression modules. The center of each graph represents *ACE2* (a) or *TMPRSS2* (b), the circles at the edges represent the top 50 genes with the highest connectivity to *ACE2* or *TMPRSS2* based on the WGCNA analysis. The circle size represents the size of each gene node in their respective modules. The arm thickness represents the relative strength of the connection to the *ACE2* or *TMPRSS2* expression. Figure was created using R 3.6 (https://www.r-project.org/)^11^.

The Lung eQTL Consortium cohort used in this gene network analysis is described in Table 1. Supplementary Fig. S1 shows the expression levels of *ACE2* and *TMPRSS2* in the three centres that are part of the Lung eQTL Consortium (see Methods); *ACE2* had low to moderate expression levels in lung tissue; whereas *TMPRSS2* was highly expressed. Based on the study cohort lung expression profile, we determined that *ACE2* and *TMPRSS2* were contained in distinct modules. The module containing *ACE2* (*ACE2* module) included 681 unique genes, while the modules containing *TMPRSS2* (*TMPRSS2* module) encompassed 1,086 unique genes. The hub gene for the *ACE2* module was *TMEM33*, and hub gene for the *TMPRSS2* module was *PDZD2* (see Methods for the definition of ‘hub gene’). Figure 1 shows the top 50 genes with the highest connectivity to *ACE2* and *TMPRSS2* within their respective modules, based on the WGCNA analysis.

**Table 1:** Study cohort demographics.

Centre	Lung eQTL consortium cohort		
	Groningen	Laval	UBC
n	342	409	287
Age, years^†^	54 (44–62)	64 (57–71)	63 (55–71)
Females, n (%)	160 (46.78)	180 (44.01)	132 (45.99)
BMI, kg/m^2†^	22.65 (20.00–25.42)	26.10 (22.90–29.00)	24.90 (22.20–28.93)
COPD^‡^, n (%)	289 (86.27)	127 (31.13)	10 (100.00)
Asthma, n (%)	0 (0.00)	15 (3.68)	22 (10.78)
Cardiac disease, n (%)	26 (7.60)	120 (29.41)	46 (21.90)
Hypertension, n (%)	2 (22.22)	107 (26.23)	33 (100.00)
Diabetes, n (%)	27 (7.89)	41 (10.05)	13 (28.26)
Never smokers, n (%)	100 (29.24)	36 (8.80)	26 (9.06)
Former smokers, n (%)	185 (54.09)	283 (69.19)	163 (56.79)
Current smokers, n (%)	57 (16.67)	90 (22.00)	98 (34.15)

^†^Median (interquartile range). ^‡^Chronic obstructive pulmonary disease. Denominators for the percentages are based on the number of non-missing records.

### *ACE2* module

We filtered out the genes with consensus module membership (MM) (see Methods) < 0.20, which left 578 genes for the subsequent analyses. The MM for *ACE2* was 0.25. We utilized genes in the *ACE2* module to execute a pathway enrichment analysis, which showed significant enrichment of two Kyoto Encyclopedia of Genes and Genomes (KEGG) pathways (‘Lysosome’, ‘Metabolic pathways’) (Supplementary Table S3) and five Gene Ontology (GO) biologic processes (*FDR* < *0.05*) (Supplementary Table S4).

### *ACE2*-correlated genes

The expression of 561 genes in the *ACE2* module was significantly correlated with *ACE2* levels (*FDR* < *0.05*), and only three of those genes were negatively correlated with *ACE2*. Although a large proportion of genes were significantly related to *ACE2* expression levels, only 78 genes had moderate or high correlations (r > 0.3).

The *PCCB* gene was most strongly correlated with *ACE2* expression (r = 0.45, Supplementary Table S1). Of the top 10 genes most strongly correlated with *ACE2*, three genes (*PCCB*, *PIGN* and *ADK*) were part of the KEGG ‘metabolic pathway’ which showed enrichment with *ACE2* module genes (Supplementary Table S3). Furthermore, out of the top 20 genes, only *WDFY3* was found in ‘process utilizing autophagic mechanism’ GO process that was enriched with *ACE2* module genes (Supplementary Table S4).

We identified 78 genes that showed moderate correlation (r > 0.3) with *ACE2* expression. Of these, 46 genes had biological and/or druggability information available (details are presented in Supplementary Table S1). We used these genes to construct a ‘map’ of biological information (Supplementary Fig. S2). Based on the druggability scores, we identified 10 genes (*GART, DPP4, PIGF, HDAC8, MDM2, ME1, SOAT1, IDE, BCAT1, ADK*) that are known drug targets by a drug compound or are targets with a known bioactive drug-like small molecule (see Methods for details on druggability scores). The number of drug-gene interactions for the 10 genes are shown in Table 2.

**Table 2:** Drug-gene interactions of *ACE2*-correlated genes.

Gene	Druggability score^†^	No. of known drug-gene interactions^‡^	r (*ACE2*)	*p*	FDR
*ADK*	Tier 2	9	0.37	*6.15* × *10*^*–36*^	5.94 × 10^–34^
*SOAT1*	Tier 2	7	0.36	*6.68* × *10*^*–34*^	3.04 × 10^–32^
*GART*	Tier 1	3	0.36	4.31 × 10^–33^	1.85 × 10^–31^
*BCAT1*	Tier 2	6	0.35	*1.16* × *10*^*–31*^	3.73 × 10^–30^
*IDE*	Tier 2	5	0.33	*6.45* × *10*^*–29*^	1.20 × 10^–27^
*MDM2*	Tier 1	20	0.33	*1.04* × *10*^*–28*^	1.79 × 10^–27^
*DPP4*	Tier 1	58	0.33	*4.99* × *10*^*–28*^	8.03 × 10^–27^
*PIGF*	Tier 1	2	0.32	*4.75* × *10*^*–26*^	5.83 × 10^–25^
*HDAC8*	Tier 1	48	0.32	*1.16* × *10*^*–25*^	1.30 × 10^–24^
*ME1*	Tier 2	2	0.31	4.40 × 10^–24^	3.86 × 10^–23^

^†^From Finan et al.^12^^‡^From Drug-Gene Interaction Database (DGIdb)^13^. r(*ACE2*): Pearson correlation coefficient between gene and *ACE2* expression (adjusted for sex age and centre). p: significance of the Pearson correlation coefficient (corresponds to Fisher’s Z score value), adjusted for false discovery rate (FDR).

### *TMPRSS2* module

*TMPRSS2* demonstrated a MM of 0.27 (Supplementary Table S2). Genes in the *TMPRSS2* module (genes with MM > 0.20) were enriched in 24 KEGG pathways (Supplementary Table S5) and 144 GO biologic processes (Supplementary Table S6). The ‘Receptor-mediated endocytosis’ GO biologic process identified in this study (*FDR* = 1.07 × 10^–04^), also contained *TMPRSS2*.

### *TMPRSS2*-correlated genes

**Figure 2:**
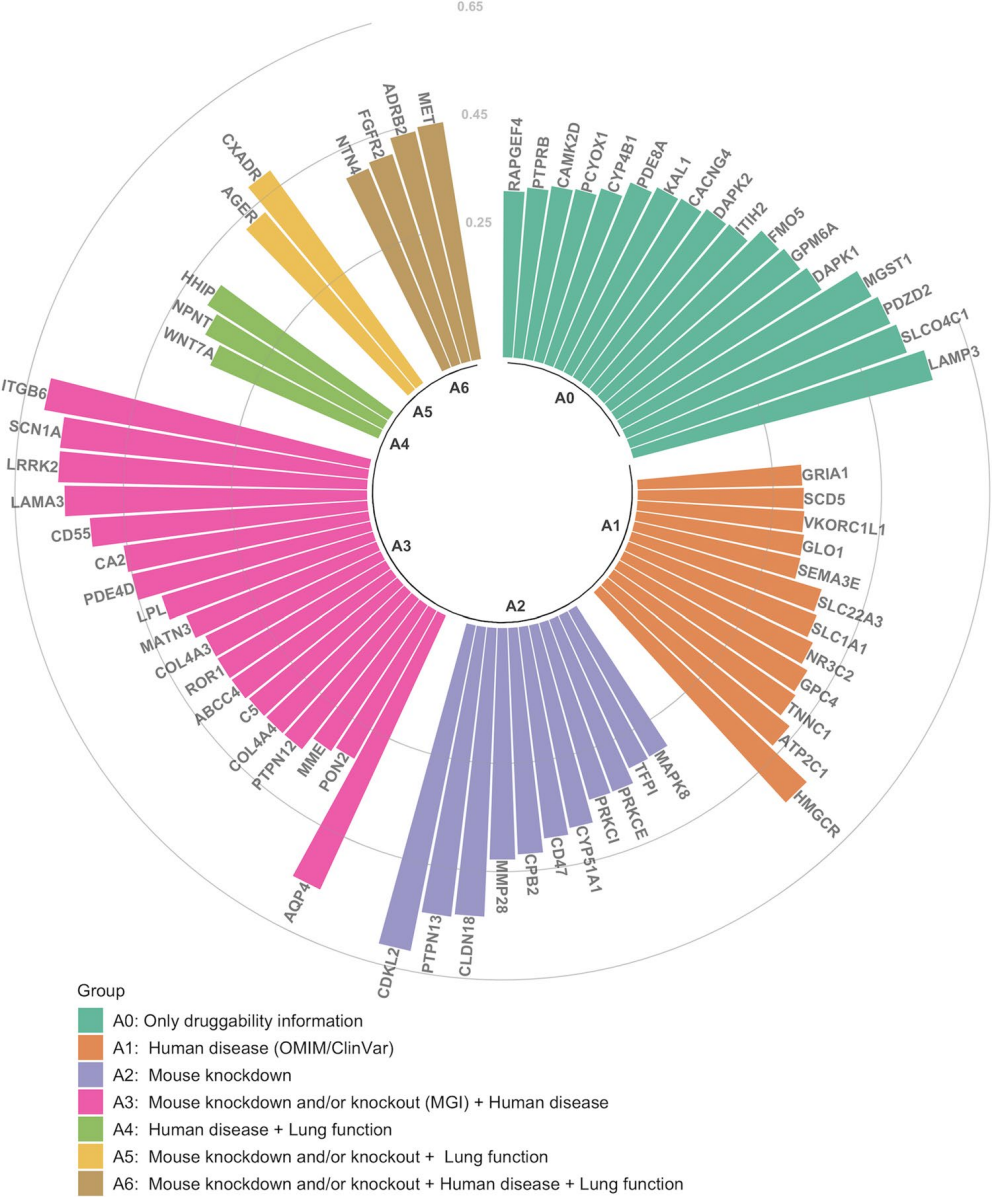
Correlation level and annotation of *TMPRSS2*-correlated genes. Each bar represents a single gene (all with druggability scores Tier 1–3^12^), and Pearson correlation coefficient (r) between the gene and *TMPRSS2* within the module is shown on the y axis. Colours of bars represent combined biological information: aqua (group A0) represents those genes with only druggability information; orange (group A1) represents genes that are related to human diseases based Online Mendelian Inheritance in Man (OMIM) and ClinVar databases; purple (group A2) represents genes with phenotypic information on knockdown or knockout mouse models based on Mouse Genome Informatics (MGI) database; pink (group A3) represents genes with phenotypic information on knockdown or knockout mouse models based on MGI database, and that are related to human diseases based OMIM and ClinVar data bases; green (group A4) represents genes related to human diseases based OMIM and ClinVar databases and with genetic variants associated to lung function traits^14^; yellow (group A5) represents genes with phenotypic information on knockdown or knockout mouse models based on MGI database, and with genetic variants associated to lung function traits^14^; brown (group A6) represents genes with phenotypic information on knockdown or knockout mouse models based on MGI database, and that are related to human diseases based OMIM and ClinVar data bases, and with genetic variants associated to lung function traits^14^. Figure was created using R 3.6 (https://www.r-project.org/)^11^.

We found that 771 unique genes (genes with MM > 0.20) in the *TMPRSS2* module were correlated with the *TMPRSS2* expression level in lung tissue (*FDR* < *0.05*), with *FHDC1* expression showing the strongest relationship with *TMPRSS2* (r = 0.72) (Supplementary Table S2). Next, we identified 325 genes that were moderately or highly correlated with *TMPRSS2* gene expression levels (r > 0.30), of these 67 were drug targets or were part of key pathways that could be targeted by drug compounds, including *TMPRSS2* (see Methods), however we focused on those that are drug targets (Table 3). The genes are shown in Fig. 2, grouped based on the availability of biological information. The A6 group contained the genes with the largest amount of biological information in the explored bioinformatics databases. Most genes in Fig. 2 had information on druggability scores, and mouse and human phenotypes (A3 group); details on the genes biological information are presented in the Supplementary Table S2.

**Table 3:** Drug-gene interactions of *TMPRSS2*-correlated genes.

Gene	No. of drug-gene interactions^‡^	r (*TMPRSS2*)	*p*	FDR
*CDKL2*	1	0.61	1.77 × 10^–116^	1.11 × 10^–114^
*ITGB6*	4	0.61	9.72 × 10^–113^	5.66 × 10^–111^
*SCN1A*	75	0.57	4.39 × 10^–95^	1.25 × 10^–93^
*LRRK2*	25	0.56	2.22 × 10^–93^	5.92 × 10^–92^
*LAMA3*	2	0.55	4.64 × 10^–89^	1.11 × 10^–87^
*HMGCR*	28	0.55	5.85 × 10^–86^	1.28 × 10^–84^
*SLCO4C1*	1	0.54	1.90 × 10^–83^	3.93 × 10^–82^
*CLDN18*	1	0.53	9.85 × 10^–82^	1.91 × 10^–80^
*MGST1*	2	0.52	4.12 × 10^–77^	6.81 × 10^–76^
*CD55*	3	0.51	6.88 × 10^–72^	1.00 × 10^–70^
*DAPK2*	1	0.47	6.68 × 10^–61^	7.62 × 10^–60^
*SLC1A1*	8	0.47	2.64 × 10^–60^	2.99 × 10^–59^
*PDE4D*	44	0.45	6.43 × 10^–55^	6.02 × 10^–54^
*CA2*	75	0.45	1.25 × 10^–54^	1.15 × 10^–53^
*ADRB2*	164	0.45	6.33 × 10^–54^	5.69 × 10^–53^
*FGFR2*	55	0.45	1.35 × 10^–53^	1.21 × 10^–52^
*MET*	118	0.44	1.11 × 10^–52^	9.48 × 10^–52^
*DAPK1*	3	0.44	3.86 × 10^–51^	3.05 × 10^–50^
*FMO5*	1	0.43	5.27 × 10^–48^	3.73 × 10^–47^
*SLC22A3*	4	0.40	1.95 × 10^–41^	1.08 × 10^–40^
*ABCC4*	30	0.40	3.30 × 10^–41^	1.80 × 10^–40^
*NR3C2*	29	0.38	4.28 × 10^–38^	2.16 × 10^–37^
*CYP51A1*	5	0.38	1.51 × 10^–37^	7.44 × 10^–37^
*PDE8A*	4	0.37	6.24 × 10^–35^	2.77 × 10^–34^
*C5*	10	0.36	4.38 × 10^–33^	1.81 × 10^–32^
*MME*	15	0.36	5.65 × 10^–33^	2.32 × 10^–32^
*CYP4B1*	2	0.34	1.08 × 10^–29^	4.00 × 10^–29^
*PRKCI*	12	0.33	2.57 × 10^–28^	9.10 × 10^–28^
*PRKCE*	16	0.33	3.55 × 10^–28^	1.24 × 10^–27^
*CAMK2D*	17	0.33	5.03 × 10^–28^	1.74 × 10^–27^
*MAPK8*	48	0.30	5.53 × 10^–24^	1.67 × 10^–23^
*GRIA1*	33	0.30	8.11 × 10^–24^	2.42 × 10^–23^

^‡^from Drug-Gene Interaction Database (DGIdb)^13^. r(*TMPRSS2*): Pearson correlation coefficient between gene and *TMPRSS2* expression (adjusted for sex, age and centre). p: significance of the Pearson correlation coefficient (corresponds to Fisher’s Z score value), adjusted for false discovery rate (FDR).

We later explored the drug-gene interactions of the genes described in Fig. 2; 52 of these genes were found to interact with known drugs. The genes with the highest druggability score (Tier 1) are presented in Table 3. Furthermore, Table 3 includes one of the genes (*ADRB2*) that is part of the GO biological process related to *TMPRSS2* (‘Receptor-mediated endocytosis’).

### Effects of clinical variables on *ACE2*- and *TMPRSS2*-correlated genes

**Figure 3:**
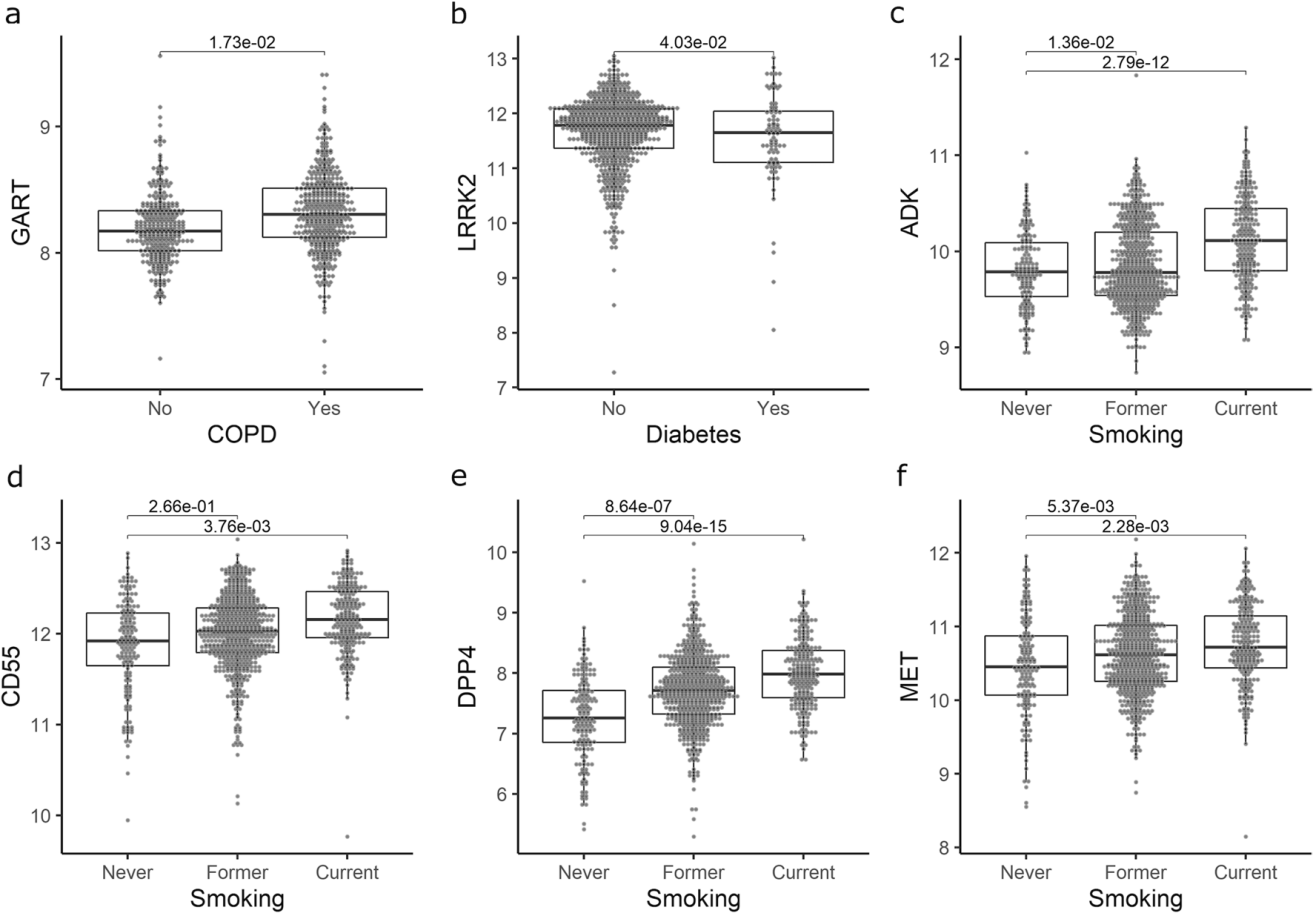
Effects of COVID-19 risk factors on lung tissue gene expression. y axes represent the microarray gene expression level in lung tissue for *ACE2*-correlated genes (*GART* [**a**]*, ADK* [**c**], *DPP4* [**e**]) and *TMPRSS2*-correlated genes (*LRRK2* [**b**]*,CD55* [**d**], *MET* [**f**]). Boxes are median expression ± interquartile range respectively. Numbers at the top of each box plot are FDR obtained from the robust linear regressions. Figure was created using R 3.6 (https://www.r-project.org/)^11^.

We investigated the effects of risk factors for COVID-19 on the expression of the genes shown in Tables 2 and 3. A full list of differential expressed genes (*FDR* < *0.05*) with known drug-gene interactions is presented in Supplementary Table S7. Some illustrative examples are shown in Fig. 3, including the effect of chronic obstructive pulmonary disease (COPD) on *GART* expression (Fig. 3a), the effect of diabetes on *LRRK2* expression (Fig. 3b), and the effect of smoking on *ADK*, *CD55, DPP4* and *MET* expression (Fig. 3c–f).

## Discussion

There is a scarcity of therapeutic treatments specific for this virus and for severe COVID-19 pneumonia. *ACE2* and *TMPRSS2* are key proteins involved in the cellular entry mechanism of SARS-CoV-2 to infect the lungs of the human host. Because one of the rate-limiting steps in this process is the overall availability of these proteins on the surface of lung epithelial cells^15^, careful evaluation of *ACE2* and *TMPRSS2* biology may enable identification of possible therapeutic targets against SARS-CoV-2 infection. In this study, by using a network analysis of genome-wide gene expression in lung tissue, we were able to identify a set of genes that interact with *ACE2* and *TMPRSS2*, and thus may be drug targets.

Other investigators have explored lung gene coexpression in order to identify potential drug targets in COVID-19^16^. Recently, Cava et al. reported genes that are co-expressed with *ACE2* using a series of pairwise correlations, and assembled the correlated genes into a protein–protein interaction network. In this way, they were able to identify 526 genes with significant (*p* < 0.01) correlation to *ACE2* and 36 existing drugs known to interact with protein-coding, *ACE2*-related genes. While this approach was a useful exploration, we believe our approach has several advantages. First, WGCNA incorporates the coordinated expression of genes and thus is more reflective of the complex regulation of gene expression. Second, our analysis controls for the false discovery rate rather than setting a nominal p value cutoff that does not correct for multiple testing. Third, we also explored coexpression with the host cell protease gene TMPRSS2, which revealed many more targets with high druggability and known drug-gene interactions than those related to *ACE2*. Finally, we used gene expression data from a large lung tissue data set with detailed clinical phenotype information, which allowed us to relate our findings to known risk factors for severe COVID-19. Despite the differences in approach, our results show some overlap with those of Cava et al.^16^—12 *ACE2*-correlated genes were identified by both approaches, suggesting that these may be some of the more robust targets for further investigation.

One notable gene that was confirmed by our study was *ADK*^16^. This gene is a key regulator of extracellular and intracellular adenine nucleotides^17,18^. ADK inhibition attenuates lung injury in mice^19^, while in humans, cigarette exposure upregulates expression of *ADK* in lung tissue. We posit a role for *ADK* in COVID-19. We believe that up-regulation of ADK expression may increase the concentration of adenosine in the lungs, which in turn can enhance viral replication. Previous work has shown that silencing *ADK* decreased influenza replication in an in vitro model^20^. Another study showed that ADK can activate didanosine^21^, a dideoxynucleoside analogue of adenosine that inhibits retro-transcription and is used in the treatment of HIV. Although this drug was recently nominated for drug repurposing as a potential treatment against COVID-19^22^, the biology of this drug is complex, particularly given the detrimental effect of ADK on lung injury.

Another *ACE2*-correlated gene that emerged from this study was *DPP4. DPP4* encodes the dipeptidyl-peptidase 4 (DPP-4) glycoprotein, which plays a major role in glucose and insulin metabolism and is linked to diabetes, now established as a key risk factor for severe COVID-19 including mortality^23^. DPP-4 is the functional receptor for the Middle East Respiratory Syndrome (MERS) coronavirus and interacts with dozens of drugs. DPP-4 inhibitors, which are used in the treatment of diabetes, appear to reduce macrophage infiltration and insulin resistance but have not been shown to increase the risk of infection in diabetic patients^24^. However, the effects of DPP-4 inhibitors on the immune response are not well understood. Because of the similarities between MERS and SARS-Cov-2, this is an interesting potential target, particularly for patients with diabetes. Thus, the DPP-4 pathway poses an intriguing possibility for novel COVID-19 therapeutics.

The *HDAC8* gene is an exciting potential target because of its role in pulmonary fibrosis (PF) and its interaction with histone deacetylase (HDAC) inhibitors. HDAC inhibitors have shown promise against fibrotic diseases^25^. The overexpression of HDACs is suggested to contribute to the process of bronchiolization in patients with IPF^26^. Viral infection increases the risk of PF^27^ and it is reported that *HDAC8* inhibition ameliorates PF^28^; moreover we found that cigarette exposure, a known risk factor for both COVID-19 and IPF, increases the expression of *HDAC8* in lung tissue. Therefore, targeting the PF mechanisms through HDAC inhibitors pose an interesting therapy to further explore.

Furthermore, the *CD55* or complement decay-accelerating factor, an inhibitor of complement activation, is one of the few genes that was part of this process. The complement system has a major role in the immune response to viruses and triggers a proinflammatory cascade^29^. CD55, which is highly expressed in lung tissue, prevents the formation of C3 convertase^30^ and therefore also inhibits the formation of C3 complement. C3-deficient mice show less respiratory dysfunction and lower levels of cytokines and chemokines in lungs in response to SARS-CoV^31^. Thus, it is possible that preventing the formation of C3 via CD55 could be beneficial in COVID-19. Fortunately, known compounds such as chloramphenicol already exist that specifically target CD55^29,32^.

As noted above, we have identified a set of genes that interact with potential therapeutic targets, which could be explored as treatments against COVID-19. The main strength of our study is the large number of lung tissue specimens with detailed clinical phenotypic data. This allowed us to not only identify genes related to *ACE2* and *TMPRSS2* expression, but also to determine the effects of various clinical factors on the lung tissue expression of these genes. However, there were limitations to this study. First, we used an *in-silico* approach to identify *ACE2* and *TMPRSS2* correlated genes, but we did not confirm these association in vivo or determine how these correlated genes physically interacted with *ACE2* and *TMPRSS2*. Second, we identified the most promising drugs based on drug-gene interactions from bioinformatic databases, but we did not validate their effects on gene and/or protein expression through in vitro experiments. Third, the lungs of our study cohort were not exposed to SARS-CoV-2, therefore it is possible that the gene expression of these key identified genes in lung tissue could be changed upon SARS-CoV-2 infection. Lastly, the cohort used for gene expression was of European ancestry and the results may not be generalizable to other ethnic groups, which is of critical importance in a global pandemic.

In summary, *ACE2* and *TMPRSS2* gene networks contained genes that could contribute to the pathophysiology of COVID-19. These findings show that computational in silico approaches can lead to the rapid identification of potential drugs, which could be repurposed as treatments against COVID-19. Given the exponential spread of COVID-19 across the globe and the unprecedented rise in deaths, such rapidity is necessary in our ongoing fight against the pandemic.

## Methods

### Lung expression quantitative trait loci (eQTL) Consortium cohort and gene expression

Using microarray, gene expression profiles of 43,466 non-control probe sets (GEO platform GPL10379) were obtained from lung tissue samples in the Lung eQTL Consortium Cohort. Briefly, samples from this cohort included whole non-tumour lung tissue samples from 1,038 participants of European ancestry who underwent surgical lung resection. Tissue samples were collected based on the Institutional Review Board guidelines at three different institutions: The University of British Columbia (UBC), Laval University and University of Groningen. This study was approved by the ethics committees within each institution. A full description of the cohort and quality controls is provided by Hao and colleagues^9^.

### Gene expression network analysis

Using the WGCNA^8^ R package, we explored gene networks correlated to *ACE2* and *TMPRSS2* in order to identify potential interactions in the Lung eQTL Consortium cohort. WGCNA clusters co-expressed genes into networks and creates “modules”, which are defined as groups of highly interconnected genes. For this analysis we identified signed consensus modules among the three centres in our study cohort. Briefly, WGCNA generated a signed coexpression matrix based on the correlation between genes, which later was transformed into an adjacent matrix by raising the coexpression to a soft threshold power (β). For our study we used a β = 6 and a minimum module size of 100 probe sets. For each probe set in the modules a ‘Module Membership’ (MM) was calculated by correlating the gene’s expression with the respective module’s expression (eigengene), i.e. the first principal component of each module gene expression profile; the gene with the highest MM was termed the ‘hub gene’. Note that the modules were determined at a gene probe level and some of the genes mapped to more than one probe; the slight overlap of genes between modules suggested heterogeneity between the gene probes, thus we did not consider these genes as relevant candidates in our study.

### Enrichment analysis and correlations of *ACE2* and *TMPRSS2* modules

Enrichment analysis of KEGG pathways and GO biological processes was performed using the R package WebGestaltR (0.4.3)^33^. This analysis was applied to the genes (with MM > 0.20) in the modules containing *ACE2* (*ACE2* module) and *TMPRSS2* (*TMPRSS2* module). Significant enrichment was established at *FDR* < *0.05.* For each gene in the *ACE2* and *TMPRSS2* modules with MM > 0.20, we determined the Pearson correlation between the expression level of the gene and that of *ACE2* or *TMPRSS2*. First the expression was adjusted for age and sex by using a robust linear module, and the residuals were extracted (for each centre separately), we then used these to calculate the correlation coefficients for the three centres separately, and then combined them using correlation meta-analysis via the R package metafor^34^. Significant correlations were set at *FDR* < *0.05* and in the downstream analyses, we focused on genes that correlated to *ACE2* or *TMPRSS2* with r > 0.30.

### Drug-gene interactions and biological information of ACE2 and TMPRSS2 correlated genes

We cross-referenced the *ACE2* and *TMPRSS2* correlated genes with the Mouse Genome Informatics (MGI), the Online Mendelian Inheritance in Man (OMIM), and the ClinVar databases in order to identify biologically relevant genes. We determined druggability scores according to methods of Finan et al.^12^. Tier 1 refers to genes that are targets of small molecules and/or biotherapeutic drugs; Tier 2 score indicates gene encoding targets with a known bioactive drug-like small molecule binding partner and ≥ 50% identity (over ≥ 75% of the sequence) with an approved drug target; and Tier 3 denotes protein coding genes with similarities to drug targets and are members of key druggable gene families. We also interrogated the Drug-Gene Interaction database (DGIdb)^13^ of the genes. DGIdb defines drug-gene interaction as a known interaction (i.e.: inhibition, activation) between a known drug compound and a target gene.

### Differential expression of *ACE2* and *TMPRSS2* correlated genes

We investigated the effects of possible risk factors for COVID-19 severity (e.g. smoking, diabetes, asthma, COPD, cardiac disease, and hypertension) on the expression of druggable genes that were correlated with *ACE2* or *TMPRSS2*. Then, the differential expression was assessed for each gene-risk factor pair by a robust linear regression using the package MASS^35^ in R, in which the dependent variable was the gene expression and the explanatory variable was the risk factor. The differential expression analysis on smoking was adjusted for sex, age and centre, and the analyses on diabetes, COPD and cardiac disease and hypertension were adjusted for sex, age, smoking status and centre. We set statistically significant differential expression *FDR* < *0.05*.

## Supplementary Information

Supplementary Tables

Supplementary Figures

## Data Availability

The full results obtained in this analysis are provided in the Supplementary Tables associated to this manuscript. Lung eQTL study data used for this study can be obtained through GEO platform accession number GSE23546 and the dbGaP Study Accession phs001745.v1.p1.
