# Supplementary material for: Gene expression network analysis provides potential targets against SARS-CoV-2
Source: Sci Rep. 2020 Dec 14;10:21863. doi: 10.1038/s41598-020-78818-w (PMC7736291; doi:10.1038/s41598-020-78818-w)
Supplement: Supplementary file 2 — Supplementary Figures [file 41598_2020_78818_MOESM2_ESM.pdf]

**Gene Expression Network Analysis Provides Potential Targets Against SARS-CoV-2**

Ana I. Hernández Cordero, Xuan Li, Chen Xi Yang, Stephen Milne, Yohan Bossé, Philippe Joubert, Wim Timens, Maarten van den Berge, David Nickle, Ke Hao, Don D. Sin

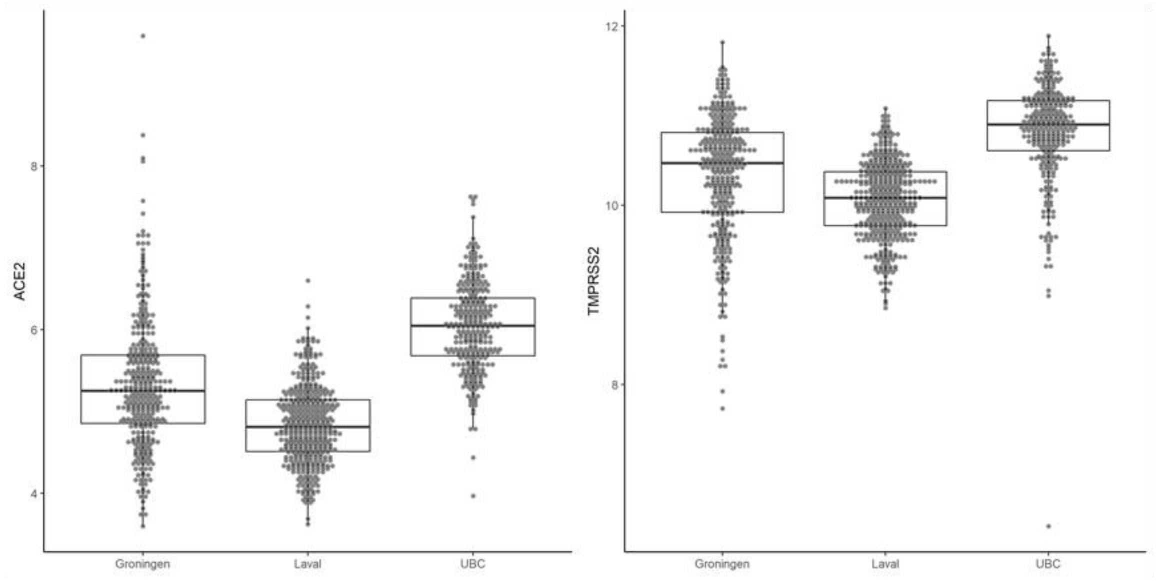


**Supplementary Figure S1.** ACE2 and TMPRSS2 expression level in lung tissue. The y-axis of the plots represents gene expression levels of ACE2 (A) and TMPRSS2 (B) and the x-axis represent each of the centres. The boxes and the horizontal line inside them, represent the interquartile range and median expression, respectively. The dots across the boxes represent the distribution of the gene expression. Figure was created using the statistical software R (https://www.r-project.org/)

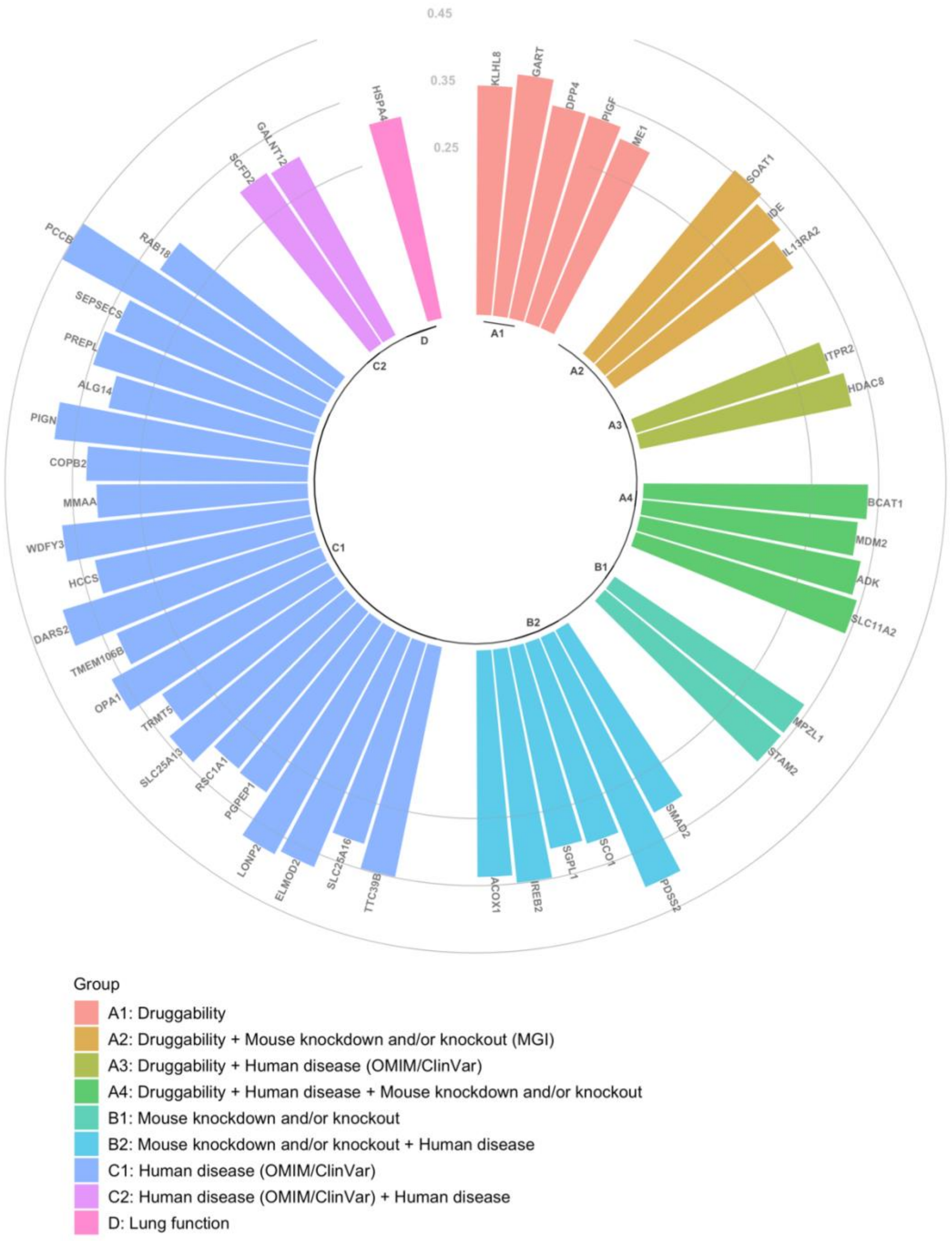


**Supplementary Figure S2.** Correlation level (y-axis) and annotation for ACE2 correlated genes. Each bar represents a single gene, and Pearson correlation coefficient (r) between the gene and TMPRSS2 within the module is shown on the y axis. Colours of bars represent combined biological information as described in the 'Group' information provided below the figure. Figure was created using the statistical software R (https://www.r-project.org/)
